# Active surveillance testing to reduce transmission of carbapenem-resistant, gram-negative bacteria in intensive care units: a pragmatic, randomized cross-over trial

**DOI:** 10.1186/s13756-023-01222-2

**Published:** 2023-03-03

**Authors:** Jiwon Jung, Joung Ha Park, Hyejin Yang, Young-Ju Lim, Eun Ok Kim, Chae-Man Lim, Mi-Na Kim, Min-Woo Jo, Sung-Cheol Yun, Sung-Han Kim

**Affiliations:** 1 Department of Infectious Disease, Asan Medical Center, University of Ulsan College of Medicine, Seoul, South Korea; 2 Division of Infectious Diseases, Department of Internal Medicine, Chung-Ang University Gwangmyeong Hospital, Gwangmyeong, South Korea; 3 Office for Infection Control, Asan Medical Center, Seoul, South Korea; 4 Department of Pulmonary and Critical Care Medicine, Asan Medical Center, University of Ulsan College of Medicine, Seoul, South Korea; 5 Department of Laboratory Medicine, Asan Medical Center, University of Ulsan College of Medicine, Seoul, South Korea; 6 Department of Preventive Medicine, University of Ulsan College of Medicine, Seoul, South Korea; 7 Department of Clinical Epidemiology and Biostatistics, Asan Medical Center, University of Ulsan College of Medicine, Seoul, South Korea

**Keywords:** Active surveillance testing, Carbapenem-resistant, Gram-negative bacteria, Contact precautions

## Abstract

**Background:**

In intensive care unit (ICU) settings, the transmission risk of carbapenem-resistant, gram-negative bacteria (CRGNB) is high. There is a paucity of data regarding the effectiveness of interventions, including active screening, preemptive isolation, and contact precautions, to reduce transmission of CRGNB.

**Methods:**

We conducted a pragmatic, cluster-randomized, non-blinded cross-over study in 6 adult ICUs in a tertiary care center in Seoul, South Korea. ICUs were randomly assigned to perform active surveillance testing with preemptive isolation and contact precautions (intervention) or standard precautions (control) during the initial 6-month study period, followed by a 1-month washout period. During a subsequent 6-month period, departments that used standard precautions switched to using interventional precautions and vice versa. The incidence rates of CRGNB were compared between the two periods using Poisson regression analysis.

**Results:**

During the study period, there were 2268 and 2224 ICU admissions during the intervention and control periods, respectively. Because a carbapenemase-producing Enterobacterales outbreak occurred in a surgical ICU (SICU), we excluded admissions to the SICU during both the intervention and control periods and performed a modified intention-to-treat (mITT) analysis. In mITT analysis, a total of 1314 patients were included. The acquisition rate of CRGNB was 1.75 cases per 1000 person-days during the intervention period versus 3.33 cases per 1000 person-days during the control period (IRR, 0.53 [95% confidence interval (CI) 0.23–1.11]; P = 0.07).

**Conclusions:**

Although this study was underpowered and showed borderline significance, active surveillance testing and preemptive isolation could be considered in settings with high baseline prevalence of CRGNB.

*Trial registration* Clinicaltrials.gov Identifier: NCT03980197.

**Supplementary Information:**

The online version contains supplementary material available at 10.1186/s13756-023-01222-2.

## Introduction

Carbapenem-resistant, gram-negative bacteria (CRGNB), including *Pseudomonas aeruginosa*, *Acinetobacter baumannii*, and Enterobacterales, have been leading causes of healthcare-associated infections and intensive care unit (ICU)-acquired infections [1]. In Korea, the proportion of carbapenem resistance rates in *A. baumannii* (CRAB) and *P. aeruginosa* (CRPA) have increased; in a 2015 surveillance program by the Korea Centers for Disease Control and Prevention, 85% of *A. baumannii* and 35% of *P. aeruginosa* were carbapenem-resistant [2]. In addition, carbapenem-resistant Enterobacterales (CRE) and carbapenemase-producing Enterobacterales have also increased exponentially [3]. Transmission of CRGNB is a great burden in hospitals because there are limited treatment options for CRGNB infections, and it has high morbidity and mortality. To prevent transmission of CRGNB, infection-control measures, including promotion of hand hygiene, environmental cleaning, and screening for carriers, have been implemented. However, there is limited evidence that screening for identification of CRGNB carriers is useful. For methicillin-resistant *Staphylococcus aureus* (MRSA), several studies found that screening and isolation were not effective for reducing its transmission [4] with good hand hygiene compliance and daily chlorhexidine-bathing. Thus, we aimed to evaluate the effectiveness of active surveillance testing for identifying CRGNB carriers to reduce its transmission in ICUs in the chlorhexidine-bathing era.

## Methods

### Study design

We conducted a pragmatic, cluster-randomized, nonblinded cross-over study in the included randomized ICUs between June 2019 and June 2020. We included 6 adult ICUs in a tertiary care hospital, Seoul, South Korea: two medical ICUs (23 beds), two surgical ICUs (26 beds), a cardiac ICU (16 beds), and a cardiothoracic surgery ICU (15 beds) in a tertiary care hospital. The study was approved by the physicians and nurse team leaders of each ICU and the institutional review board (IRB no. 2019–0274). The requirements for informed consent were waived. ICUs were randomly assigned to perform active surveillance testing (intervention) or use standard precautions (control) during the initial 6-month study period (period 1), followed by a 1-month washout period, and alternative during the second 6-month period (period 2). Randomization of ICU was performed by SPSS for Windows software, version 21 (SPSS Inc., Chicago, IL, USA). The microbiology laboratory processed surveillance specimens using standard culture-based identification of CRGNB. Patients with histories of CRGNB colonization or infection were placed under contact precautions at the time of admission.

### Active surveillance and contact precautions

In the intervention period, stool or perirectal swabs for CRPA, CRAB, and CRE surveillance cultures and sputum, or endotracheal cultures for CRPA or CRAB, were obtained from patients within 2 days of their admission to the ICU and weekly thereafter. In the intervention period, preemptive isolation and contact precautions were implemented at admission, and if the initial surveillance test was negative, contact precautions were ceased, and standard precautions were continued. If the initial surveillance test or subsequent surveillances or clinical culture tests were positive for CRGNB, isolation and contact precautions were continued until 3 negative consecutive test results were obtained. In the control period, surveillance testing was not performed, and if clinical specimens were positive for CRGNB, contact precautions were implemented. During both the intervention and control periods, daily chlorhexidine-bathing was performed in all ICUs, and contact precautions were required in patients with MRSA and VRE colonization or infection. In period 2 (from April to June 2020), universal use of personal protective equipment (PPE) (gown, glove, KF94 mask, and face shield or goggle) was implemented for response to COVID-19 pandemic when caring patients in ICUs. During the whole study period, hand hygiene compliance was observed 4 times by a year by the infection control team staff, and the results by units were disclosed to all hospital staffs. Promotions for improving the compliance of hand hygiene included frequent monitoring and real-time feedback by infection control leader in ICU nursing team, and hospital-wide rewards given to the units with high hand hygiene compliance.

If outbreaks of CRGNB occurred, surveillance and post-outbreak surveillance in the control period were permitted.

### Definition

An event was defined as a positive result for CRGNB from a clinical culture. The event date was the date of the earliest positive clinical culture. A patient was classified as having a new event if they had stayed in the ICU > 2 days, had no history of colonization or infection during the previous year, had no positive clinical culture within 2 days after admission to the ICU, and if admitted to an intervention ICU, a negative surveillance culture was obtained within 2 days of admission. Days at risk were calculated from the date of the third day in the ICU through the event date or the date two days after discharge from the ICU, whichever was later.

The primary outcome was the ICU-level incidence of new events per 1000 ICU patient-days at risk. Secondary ICU-level outcomes were the incidences of new events with CRPA, CRAB, or CRE calculated separately and the incidences of hospital-acquired bloodstream infections, catheter-related bloodstream infections, urinary tract infections, catheter-associated urinary tract infections, pneumonia, ventilator-associated pneumonia, and in-ICU mortality. We also performed subgroup analysis of individual ICUs for new events per 1000 ICU patient-days at risk. In addition, we compared new events per 1000 ICU patient-days at risk between periods 1 and 2. For the evaluation of economic impacts, we also compared the lengths of hospital and ICU stays and the costs of hospitalization between the intervention and control periods.

Outbreak was defined as ≥ 3 cases of acquisition of CRGNB within 2 weeks. If surveillance and post-outbreak surveillance were performed in the control period because of a CRGNB outbreak, we excluded the ICU in the modified intention-to-treat (mITT) analysis.

### Statistical analysis

Based on the acquisition rate of CRGNB from 2016 to 2018 in ICUs of our hospital, we assumed a mean baseline incidence of CRGNB colonization or infection of 8 per 1000 patient-days; between-cluster variance would be 0.4, and the average amount of time a patient spent in the ICU would be 10 days. This study was designed to achieve 80% power for detecting a reduction in acquisition of 40% in the intervention period with a 2-sided type I error of 5%. According to these assumptions, the estimated sample size was 2400 patients (200 per cluster; a total of 12 clusters with one cross-over of 6 ICUs) [5].

Categorical variables were analyzed using the chi-square or Fisher’s exact test, as appropriate. Normally and non-normally distributed continuous variables were analyzed by Student’s *t* test and the Mann–Whitney U test, respectively. The primary analysis was a comparison of the primary outcomes between the intervention and control periods using an unadjusted Poisson regression model according to the mITT. All statistical analyses were performed using SPSS for Windows software, version 21 (SPSS Inc., Chicago, IL, USA) and MedCalc Statistical Software version 18.10.2 (MedCalc Software bvba, Sotend, Belgium) with P < 0.05 considered statistically significant.

## Results

### Characteristics of ICUs and patients

**Fig. 1:**
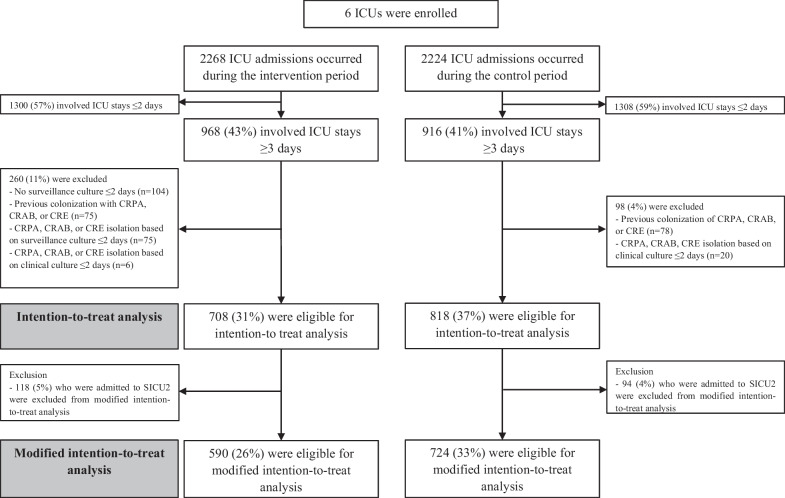
Schematic flow chart of the study. *ICU* intensive care unit; *CRPA* carbapenem-resistant *P. aeruginosa*; *CRAB* carbapenem-resistant *A. baumannii*; *CRE* carbapenem-resistant Enterobacterales

A total of 4492 admissions to the 6 ICUs occurred during the study period, and 1884 (42%) with ICU stays ≥ 3 days were enrolled in this study (Fig. 1). Two hundred and sixty patients in the intervention period and 98 patients in the control period were excluded for ITT analysis, respectively. A CRE outbreak occurred in SICU2 during the intervention period, and post-outbreak surveillance of CRE was performed in the control period; thus, we excluded the 212 patients admitted to SICU2 from the mITT analysis. The original and revised study designs are shown in Additional file 1: Figure S1. There were no significant differences in characteristics between patients in the intervention period and those in the control period (Table 1). During the total study period, the observed hand hygiene compliance was 96%. The number of clinical specimens submitted to the laboratory was not different between intervention and control periods in mITT analysis (mean [IQR], 3634 [2824–5568] in intervention period vs. 2767 [1902–4378] in control period; P = 0.35).

**Table 1:** Demographic and baseline characteristics of the study population

	Intervention period (n = 590)	Control period (n = 724)	*P* value
Male sex	338 (57.3)	439 (60.6)	0.22
Age, mean ± SD	66.1 ± 13.3	65.8 ± 13.5	0.69
Underlying diseases			
Solid cancer	143 (24.2)	183 (25.3)	0.66
Hematologic malignancy	33 (5.6)	47 (6.5)	0.46
Solid organ transplant	34 (5.8)	49 (6.8)	0.46
Hematopoietic stem cell transplant	9 (1.5)	15 (2.1)	0.46
End-stage renal disease, on dialysis	33 (5.6)	36 (5.0)	0.62
Antibiotics used within the previous 3 months			
Cefazolin	73 (12.4)	79 (10.9)	0.41
3rd cephalosporin	95 (16.1)	143 (19.8)	0.09
Piperacillin/tazobactam	177 (30.0)	198 (27.3)	0.29
Fluoroquinolone	168 (28.5)	198 (27.3)	0.65
Carbapenem	90 (15.3)	120 (16.6)	0.52
Glycopeptide (vancomycin or teicoplanin)	113 (19.2)	142 (19.6)	0.83
Other	180 (30.5)	210 (29.0)	0.55
ICU stay, days (mean ± SD)	11.0 ± 12.0	11.2 ± 13.7	0.73

The data are shown as no. (%) unless otherwise indicated

*SD* standard deviation

### Results of the acquisition rate of CRGNB according to clinical culture

In the mITT analysis, the acquisition rate of CRGNB was 1.75 cases per 1,000 person-days in the intervention period versus 3.33 cases per 1000 person-days in the control period (incidence rate ratio [IRR], 0.53; 95% confidence interval [CI] 0.23–1.11; P = 0.07) (Table 2).

**Table 2:** Acquisition rates of CRPA, CRAB, and CRE in clinical specimens between the intervention and control periods

	Intervention period, per 1000 person-days (95% CI)	Control period, per 1000 person-days (95% CI)	Incidence rate ratio (95% CI)	*P* value
*Modified intention-to-treat analysis*^a^				
Total	1.75 (0.87–3.13)	3.33 (2.16–4.92)	0.53 (0.23–1.11)	0.07
CRPA	0.32 (0.04–1.15)	1.07 (0.46–2.10)	0.30 (0.03–1.50)	0.10
CRAB	0.80 (0.26–1.86)	1.73 (0.92–2.96)	0.46 (0.13–1.37)	0.13
CRE	0.80 (0.26–1.86)	0.93 (0.38–1.92)	0.85 (0.21–3.12)	0.79

*CRPA* carbapenem-resistant *P. aeruginosa*; *CRAB* carbapenem-resistant *A. baumannii*; *CRE* carbapenem-resistant Enterobacterales; *CI* confidence interval

^a^Excluding SICU2 in both periods 1 and 2

### Secondary outcomes

There were no significant differences in the acquisition rates of CRPA, CRAB, and CRE in the intervention and control periods (CRPA, 0.32 vs. 1.07 per 1,000 person-days; IRR, 0.30 [95% CI 0.03–1.50]; P = 0.10; CRAB, 0.80 vs. 1.73 per 1000 person-days; IRR, 0.46 [95% CI 0.13–1.37]; P = 0.13; CRE, 0.80 vs. 0.93; IRR, 0.85 [95% CI 0.21–3.12]; P = 0.79) (Table 2). In addition, there were no significant differences in the rates of hospital-acquired bloodstream infections, catheter-related bloodstream infections, urinary tract infections, catheter-associated urinary tract infections, pneumonia, ventilator-associated pneumonia, and in-ICU mortality (Table 3).

**Table 3:** Clinical manifestations and outcomes between the intervention and control periods

	Intervention period (n = 590)	Control period (n = 724)	*P* value
Clinical diagnosis of infectious diseases			
Hospital-acquired bloodstream infection	0	1 (0.1)	0.37
CRPA	0	0	–
CRAB	0	1 (0.1)	0.37
CRE	0	0	–
Catheter-related bloodstream infection	0	1 (0.1)	0.37
CRPA	0	0	–
CRAB	0	1 (0.1)	0.37
CRE	0	0	-
Urinary tract infection	0	1 (0.1)	0.37
CRPA	0	0	-
CRAB	0	0	-
CRE	0	1 (0.1)	0.37
Catheter-associated urinary tract infection	0	1 (0.1)	0.37
CRPA	0	0	–
CRAB	0	0	–
CRE	0	1 (0.1)	0.37
Pneumonia	0	4 (0.6)	0.07
CRPA	0	2 (0.3)	0.20
CRAB	0	2 (0.3)	0.20
CRE	0	1 (0.1)	0.37
Ventilator-associated pneumonia	0	3 (0.4)	0.12
CRPA	0	2 (0.3)	0.20
CRAB	0	1 (0.1)	0.37
CRE	0	1 (0.1)	0.37
Death			–
In-ICU mortality^a^	70 (11.9)	76 (10.5)	0.43

*CRPA* carbapenem-resistant *P. aeruginosa*; *CRAB* carbapenem-resistant *A. baumannii*; *CRE* carbapenem-resistant Enterobacterales

The data are shown as no. (%) unless otherwise indicated

^a^Death during the follow-up periods (until 2 days after ICU discharge)

The subgroup analysis of the CRGNB acquisition rate by ICU is shown in Additional file 1: Table S1. The acquisition rates of CRGNB were significantly higher in the control period than in the intervention period in MICU2, SICU1, and the cardiac ICU, while the rates were higher in the intervention period than in the control period in MICU1 and SICU2; there was no difference between the rates in the intervention and control periods in the cardiothoracic surgery ICU. In ITT analysis, the acquisition rate of CRGNB was 2.94 cases per 1000 person-days in the intervention period versus 3.46 cases per 1000 person days in the control period (IRR, 0.85; 95% CI 0.46–1.54; P = 0.56) (Additional file 1: Table S2).

The acquisition rate of CRGNB was significantly higher in period 1 than in period 2 (3.68 cases per 1000 person-days vs. 0.52 cases per 1000 person-days; P < 0.001) (Additional file 1: Table S3).

Of 104 patients who admitted to ICU during the intervention period but did not perform surveillance culture within 2 days after ICU admission, 15 were admitted to SICU2. We compared the baseline characteristics of the remaining 89 patients and those enrolled in intervention group of mITT analysis (n = 590) (Additional file 1: Table S4). Solid organ transplant recipient (16.9% vs. 5.8%, P < 0.001) and patients with end-stage renal disease (12.4% vs. 5.6%, P = 0.01) were more common in patients without surveillance culture than in intervention group of mITT analysis. Patients with solid cancer was less common in those without surveillance culture than in intervention group of mITT analysis (12.4% vs. 24.2%, P = 0.01). The type of ICU was significantly different between two groups (P < 0.001), which reflects the difference of compliance of study protocol by ICUs.

In mITT analysis, 39 (7%) cases in intervention period were detected in surveillance culture. Of these, 31 were detected in surveillance culture only, and 8 were detected in both surveillance and clinical culture. Five were detected in surveillance culture earlier than in clinical culture. Therefore, 36 (6%) were actually detected in surveillance culture (only or earlier than in clinical culture).

### Evaluation of the economic impact of active surveillance testing

For the economic impact evaluation of active surveillance testing of CRGNB, we compared the lengths of hospital and ICU stays and the total cost of hospitalization between the intervention and control periods in the mITT population (Table 4). The mean length of hospital stays was 0.9 day shorter in the intervention period than in the control period, but the difference was not statistically significant (P = 0.73). Although there was an additional cost of **$**5666 for hospitalization in the intervention period than in the control period, the cost difference was also not statistically significant (P = 0.43).

**Table 4:** Lengths of hospital and ICU stays and cost of hospitalizations in the intervention and control periods (mITT population)

	Intervention period (n = 590)	Control period (n = 724)	*P* value
Length of hospital stay, mean (± SE) days	44.7 (1.9)	45.6 (1.9)	0.73
Length of ICU stay, mean (± SE) days	11.0 (0.5)	11.2 (0.5)	0.73
Cost of hospitalization ($), mean (± SE)	93,491 (6034)	87,825 (4,252)	0.43

## Discussion

This pragmatic, cluster-randomized, cross-over study showed that active surveillance testing to identify patients colonized with CRGNB was associated with non-statistically significant decrease in the acquisition of CRGNB in clinical specimens.

Early detection of patients colonized or infected with CRGNB is important for implementing timely interventions to prevent subsequent spread. However, active surveillance testing is a complicated and resource-intensive intervention that has the potential for several adverse consequences, including reduced contact between healthcare workers and patients due to contact precautions [6]. Previous studies have reported that active surveillance testing in combination with contact precautions for colonized patients contributed to the decline of MRSA or vancomycin-resistant *Enterococcus* [7–9]. However, there is limited evidence that active surveillance testing is associated with reducing CRGNB transmission, but many hospitals have implemented active surveillance testing for identifying CRGNB. Recent studies showed that screening and isolation of colonized patients do not reduce multidrug-resistant bacteria, especially MRSA, when compliance with hand hygiene and chlorhexidine-bathing is high [10–13]. We conducted this study to provide evidence of the effectiveness of active surveillance in the chlorohexidine-bathing and high hand hygiene compliance era. Our study had low power because only 79% (1884/2400) of patients were enrolled in the target sample size, and the baseline acquisition rate was lower than expected. Although it had borderline significance, we showed that about half of CRGNB acquisition can be prevented through active surveillance testing. Therefore, this strategy may be beneficial in high-baseline-prevalence settings. A large, multicenter study is needed to confirm our findings.

The incidence of CRGNB acquisition was higher in the period 1 (before COVID-19 pandemic) than in period 2 (after COVID-19 pandemic). The lower acquisition rate in period 2 may be associated with additional infection prevention measure to respond COVID-19 pandemic, especially universal donning of gown and glove. Previous cluster randomized study showed that universal glove and gown use was associated with decrease in acquisition of antibiotic-resistant gram-negative bacteria, although it was not statistically significant [14]. As we excluded SICU2 in the analysis, more patients were allocated to the control group in the first period, and we may overestimate the positive effect of the intervention. Further study for evaluating the effectiveness of active surveillance in the setting of identical infection prevention measures between intervention and control period is needed.

In our subgroup analysis, the CRGNB acquisition rate was higher in the intervention period than in the control period in MICU1 and SICU2. Although a CPE outbreak occurred in SICU2, there was no outbreak of CRGNB in MICU1, and the reason for this unanticipated finding is unclear. It may be a seasonal effect, or more enhanced environmental cleaning might have been performed during the control period due to the COVID-19 pandemic, which occurred in the latter 6-month period (Additional file 1: Table S3).

The acquisition rates of CRPA, CRAB, and CRE did not differ between the intervention and control periods. Because this trial was designed to identify the effectiveness of reducing the total CRGNB acquisition rate, further study is needed to identify the effectiveness of active surveillance testing for each organisms.

Our study has some limitations. First, it was a single-center study with a low prevalence of CRGNB. A multicenter study with variable prevalences of CRGNB is warranted to generalize our findings. Despite this limitation, well-monitored infection control practices and policies to minimize unmeasured confounding factors by different centers during the study period is a strength of our study. Second, as this study was not blinded, the difference in the number of clinical specimens submitted to the laboratory may be present, and this may have affected the chance to detect CRGNB. However, the number of clinical specimen was not different between intervention and control periods in mITT analysis. Third, we did not perform surveillance testing during the control period, which may have biased our findings. However, we performed a pragmatic trial that reflects actual clinical practices, and we evaluated the outcomes of CRGNB acquisition in clinical specimens. Fourth, data regarding immunosuppressant use was absent. Use of immunosuppressant is associated with exposure to antimicrobials and acquisition of MDR gram-negative organism [15–17]. However, there was no significant difference of the recent antibiotics exposure between the intervention period and the control period. Therefore, this limitation may not substantially affect our main findings. Finally, we performed a conventional culture method for active surveillance, and the turnaround time is longer than rapid PCR testing. Therefore, further study to evaluate active surveillance testing using PCR testing is needed.

## Conclusions

In conclusion, active surveillance testing for CRGNB may reduce its acquisition in clinical specimens in the ICU without additional costs. Individual hospitals should consider the cost-effectiveness of the intervention based on the baseline acquisition rate of CRGNB and the cost of intervention when they decide whether to adopt active surveillance testing.

## Supplementary Information

**Additional file 1**. Supplemental Figure and Tables.

## Data Availability

Data and materials are not available.
