# Supplementary material for: Active surveillance testing to reduce transmission of carbapenem-resistant, gram-negative bacteria in intensive care units: a pragmatic, randomized cross-over trial
Source: Antimicrob Resist Infect Control. 2023 Mar 3;12:16. doi: 10.1186/s13756-023-01222-2 (PMC9983515; doi:10.1186/s13756-023-01222-2)
Supplement: Supplementary file 1 — Additional file 1. Supplemental Figure and Tables. [file 13756_2023_1222_MOESM1_ESM.docx]

**Supplementary Figure 1. Study design of this pragmatic, cluster-randomized, cross-over, controlled study**

**A) Original study design**

6 ICUs (MICU1, MICU2, SICU1, SICU2, CCU, and CSICU)

Intervention^*^

(Active surveillance)

**MICU1**

Control^**^

**MICU2**

Intervention

(Active surveillance)

**SICU2**

Control

**SICU1**

Intervention

(Active surveillance)

**CSICU**

Control

**CCU**

**Period 1**

**(6 months)**

Control

**MICU1**

Intervention

(Active surveillance)

**MICU2**

Intervention

(Active surveillance)

**SICU1**

Control

**CSICU**

Intervention

(Active surveillance)

**CCU**

**Period 2**

**(6 months)**

**After the 1-month**

**washout period**

Control

**SICU2**

ICU, intensive care unit; MICU, medical intensive care unit; SICU, surgical intensive care unit; CCU, coronary care unit; CSICU, cardiac surgical intensive care unit

^*^Intervention: Daily chlorohexidine gluconate bathing plus active surveillance and contact precautions until the results of active surveillance have been obtained

^**^Control: Daily chlorohexidine gluconate bathing only

**B) Revised study design**

6 ICUs (MICU1, MICU2, SICU1, SICU2, CCU, and CSICU)

Intervention

(Active surveillance)

**MICU1**

Control

**MICU2**

Intervention

(Active surveillance)

**SICU2**

Control

**SICU1**

Intervention

(Active surveillance)

**CSICU**

Control

**CCU**

**Period 1**

**(6 months)**

Control

**MICU1**

Intervention

(Active surveillance)

**MICU2**

Intervention

(Active surveillance)

**SICU1**

Control

**CSICU**

Intervention

(Active surveillance)

**CCU**

**Period 2**

**(6 months)**

**After the 1-month**

**washout period**

Control but CRE

active surveillance

due to CRE outbreak

**SICU2^*^**

^*^Although SICU2 was originally assigned to the control group in period 2, this ICU continued to perform active surveillance for CRE due to a CRE outbreak.

|  | **Intervention period,**  **per 1,000 person-days (95% CI)** | | **Control period,**  **per 1,000 person-days (95% CI)** | | ***P* value** |
| --- | --- | --- | --- | --- | --- |
| **MICU1** | | | | | |
| Total | 7/1,477 | 4.74 (1.91–9.77) | 1/1,797 | 0.56 (0.01–3.10) | 0.02 |
| CRPA | 2/1,477 | 1.14 (0.16–4.89) | 0/1,797 | 0 (0–2.10) | 0.12 |
| CRAB | 2/1,477 | 1.17 (0.14–4.23) | 1/1,797 | 0.56 (0.01–3.10) | 0.45 |
| CRE | 4/1,477 | 2.71 (0.74–6.93) | 0/1,797 | 0 (0–2.10) | 0.03 |
| **MICU2** | | | | | |
| Total | 0/1,286 | 0 (0–2.90) | 4/1,559 | 2.57 (0.70–6.57) | 0.07 |
| CRPA | 0/1,286 | 0 (0–2.90) | 1/1,559 | 0.64 (0.02–3.57) | 0.36 |
| CRAB | 0/1,286 | 0 (0–2.90) | 1/1,559 | 0.64 (0.02–3.57) | 0.36 |
| CRE | 0/1,286 | 0 (0–2.90) | 3/1,559 | 1.92 (0.40–5.62) | 0.12 |
| **SICU1** |  |  |  |  |  |
| Total | 2/1,163 | 1.72 (0.21–6.21) | 12/1,226 | 9.79 (5.06–17.10) | 0.01 |
| CRPA | 0/1,163 | 0 (0–3.20) | 5/1,226 | 4.08 (1.32–9.52) | 0.03 |
| CRAB | 2/1,163 | 1.72 (0.21–6.21) | 6/1,226 | 4.89 (1.80–10.65) | 0.18 |
| CRE | 1/1,163 | 0.86 (0.02–4.79) | 4/1,226 | 3.26 (0.89–8.35) | 0.20 |
| **SICU2** |  |  |  |  |  |
| Total | 10/874 | 11.44 (5.49–21.04) | 4/876 | 4.57 (1.24–11.69) | 0.11 |
| CRPA | 2/874 | 2.29 (0.28–8.27) | 0/876 | 0 (0–4.20) | 0.16 |
| CRAB | 3/874 | 3.43 (0.71–10.03) | 0/876 | 0 (0–4.20) | 0.08 |
| CRE | 5/874 | 5.72 (1.86–13.35) | 4/876 | 4.57 (1.24–1.69) | 0.74 |
| **CCU** |  |  |  |  |  |
| Total | 0/1,122 | 0 (0–3.30) | 7/1,756 | 3.99 (1.60–8.21) | 0.03 |
| CRPA | 0/1,122 | 0 (0–3.30) | 2/1,756 | 1.14 (0.14–4.11) | 0.26 |
| CRAB | 0/1,122 | 0 (0–3.30) | 5/1,756 | 2.85 (0.93–6.65) | 0.07 |
| CRE | 0/1,122 | 0 (0–3.30) | 0/1,756 | 0 (0–2.10) | >0.99 |
| **CSICU** |  |  |  |  |  |
| Total | 2/1,234 | 1.62 (0.20–5.86) | 1/1,165 | 0.86 (0.02–4.78) | 0.60 |
| CRPA | 0/1,234 | 0 (0–3.00) | 0/1,165 | 0 (0–3.20) | >0.99 |
| CRAB | 1/1,234 | 0.81 (0.02–4.52) | 0/1,165 | 0 (0–3.20) | 0.33 |
| CRE | 1/1,234 | 0.81 (0.02–4.52) | 1/1,165 | 0.86 (0.02–4.78) | 0.94 |

**Supplemental Table 1. Subgroup analysis of acquisition rates of CRPA, CRAB, and CRE on clinical specimens between the intervention and control periods, stratified by ICUs**

**Supplementary Table 2. Acquisition rates of CRPA, CRAB, and CRE in clinical specimens between the intervention and control periods in intention-to-treat analysis and modified intention-to-treat analysis**

|  | **Intervention period,**  **per 1,000 person-days (95% CI)** | | **Control period,**  **per 1,000 person-days (95% CI)** | | **Incidence rate ratio (95% CI)** | ***P* value** |
| --- | --- | --- | --- | --- | --- | --- |
| **Intention-to-treat analysis** | | | | | | |
| Total | 21/7,156 | 2.94 (1.82–4.49) | 29/8,379 | 3.46 (2.32–4.97) | 0.85 (0.46–1.54) | 0.56 |
| CRPA | 4/7,156 | 0.56 (0.15–1.43) | 8/8,379 | 0.95 (0.41–1.88) | 0.59 (0.13–2.19) | 0.38 |
| CRAB | 8/7,156 | 1.12 (0.48–2.20) | 13/8,379 | 1.55 (0.83–2.65) | 0.72 (0.26–1.88) | 0.46 |
| CRE | 10/7,156 | 1.40 (0.67–2.57) | 11/8,379 | 1.31 (0.66–2.35) | 1.06 (0.40–2.76) | 0.89 |
| **Modified intention-to-treat analysis^a^** | | | | |  |  |
| Total | 11/6,282 | 1.75 (0.87–3.13) | 25/7,503 | 3.33 (2.16–4.92) | 0.53 (0.23–1.11) | 0.07 |
| CRPA | 2/6,282 | 0.32 (0.04–1.15) | 8/7,503 | 1.07 (0.46–2.10) | 0.30 (0.03–1.50) | 0.10 |
| CRAB | 5/6,282 | 0.80 (0.26–1.86) | 13/7,503 | 1.73 (0.92–2.96) | 0.46 (0.13–1.37) | 0.13 |
| CRE | 5/6,282 | 0.80 (0.26–1.86) | 7/7,503 | 0.93 (0.38–1.92) | 0.85 (0.21–3.12) | 0.79 |

CRPA, carbapenem-resistant *P. aeruginosa*; CRAB, carbapenem-resistant *A. baumannii*; CRE, carbapenem-resistant Enterobacteriaceae

^a^Excluding SICU2 in both periods 1 and 2

**Supplementary Table 3.** **Acquisition rates of CRPA, CRAB, and CRE in clinical specimens between periods 1 and 2 (modified intention-to-treat analysis population).**

|  | **Period 1,**  **per 1,000 person-days (95% CI)** | | **Period 2,**  **per 1,000 person-days (95% CI)** | | ***P* value** |
| --- | --- | --- | --- | --- | --- |
| Total | 32/8,690 | 3.68 (2.52–5.20) | 4/7,723 | 0.52 (0.14–1.33) | <0.001 |
| CRPA | 10/8,690 | 1.12 (0.55–2.12) | 0/7,723 | 0 (0–0.50) | 0.003 |
| CRAB | 15/8,690 | 1.73 (0.97–2.85) | 3/7,723 | 0.39 (0.08–1.14) | 0.01 |
| CRE | 11/8,690 | 1.27 (0.63–2.23) | 1/7,723 | 0.13 (0–0.72) | 0.007 |

**Supplemental Table 4. Demographic and baseline characteristics of patients without surveillance culture within 2 days after ICU admission and those in intervention group.**

|  | **No surveillance culture (n=89)** | **Intervention period (n=590)** | ***P* value** |
| --- | --- | --- | --- |
| **Male sex** | 60 (67.4) | 338 (57.3) | 0.07 |
| **Age, mean ± SD** | 65.8 ± 13.8 | 66.1 ± 13.3 | 0.85 |
| **Underlying diseases** |  |  |  |
| Solid cancer | 11 (12.4) | 143 (24.2) | 0.01 |
| Hematologic malignancy | 0 (0) | 33 (5.6) | 0.02 |
| Solid organ transplant | 15 (16.9) | 34 (5.8) | <0.001 |
| Hematopoietic stem cell transplant | 0 (0) | 9 (1.5) | 0.61 |
| End-stage renal disease, on dialysis | 11 (12.4) | 33 (5.6) | 0.02 |
| **Antibiotics used within the previous 3 months** |  |  |  |
| Cefazolin | 11 (12.4) | 73 (12.4) | >0.99 |
| 3^rd^ cephalosporin | 21 (23.6) | 95 (16.1) | 0.08 |
| Piperacillin/tazobactam | 29 (32.6) | 177 (30.0) | 0.62 |
| Fluoroquinolone | 20 (22.5) | 168 (28.5) | 0.24 |
| Carbapenem | 17 (19.1) | 90 (15.3) | 0.35 |
| Glycopeptide (vancomycin or teicoplanin) | 24 (27.0) | 113 (19.2) | 0.09 |
| Other | 33 (37.1) | 180 (30.5) | 0.21 |
| **ICU stay, days (mean ± SD)** | 8.5 ± 13.8 | 11.0 ±12.0 | 0.08 |
| **Type of ICU** |  |  | <0.001 |
| MICU1 | 1 (1.0) | 116 (16.4) |  |
| CSICU | 23 (22.1) | 178 (25.1) |  |
| MICU2 | 7 (6.7) | 112 (15.8) |  |
| CCU | 45 (43.3) | 112 (15.8) |  |
| SICU1 | 13 (12.5) | 72 (10.2) |  |
